# Beyond motor: a systematic review of multisensory integration deficits in Parkinson’s disease

**DOI:** 10.1007/s00702-026-03184-2

**Published:** 2026-05-29

**Authors:** Zahra Azizi, Annalisa Setti, Veronica Bruno, Jochen H. M. Prehn, Ali Idri, Tomas Ward

**Affiliations:** 1 Dublin City University, Dublin, Ireland; 2 Insight Research Ireland Centre for Data Analytics, Dublin, Ireland; 3 School of Applied Psychology, University College Cork, Cork, Ireland; 4 Department of Clinical Neurosciences, University of Calgary, Hotchkiss Brain Institute, Calgary, AB Canada; 5 Department of Physiology and Medical Physics, Royal College of Surgeons in Ireland, Dublin, Ireland; 6 FutureNeuro Research Ireland Centre, Royal College of Surgeons in Ireland, Dublin, Ireland; 7 Data and Software Sciences Research Laboratory, ENSIAS, Mohammed V University, Rabat, Morocco

**Keywords:** Parkinson's disease (PD), Multisensory integration (MSI), Postural instability, Perceptual integration, Non-motor symptoms

## Abstract

Parkinson's disease (PD) is primarily characterized by motor symptoms, but non-motor issues, including sensory, perceptual, and cognitive impairment, common, significantly impacting daily functioning. Multisensory integration (MSI), the brain's ability to combine sensory information, is crucial for effective navigation and interaction with the world. In PD, disruptions in MSI can lead to various functional difficulties. This systematic review, conducted by PRISMA guidelines, analyses 40 studies with 2070 participants to investigate MSI in PD. The findings indicate significant deficits in MSI, especially in audiovisual temporal discrimination, body ownership perceptions, and visual-vestibular integration. These deficits negatively affect movement accuracy and verticality perception. The review highlights a strong focus on balance and postural control, with multiple studies reporting issues in visual-vestibular integration and vestibular dysfunction. These impairments, along with cholinergic deficits, contribute to postural instability and increased fall risk in individuals with PD. Studies using virtual reality and portable monitors further emphasize the role of vestibular and proprioceptive dysfunction in maintaining balance. Perceptual integration is also disrupted, with altered body ownership perceptions and impaired audiovisual processing manifesting at the perceptual level, linked to subcortical structures, such as the subthalamic nucleus. These deficits extend to motor imagery and body representation, affecting both hand movement accuracy and visual-vestibular perception. This review underscores the critical role of MSI deficits in PD, highlighting the importance of understanding sensory integration in the management of the disease. Understanding these deficits can lead to more precise diagnostic tools and highly targeted rehabilitation strategies, thereby improving patient outcomes and guiding clinical practice.

**Supplementary Information:**

The online version contains supplementary material available at 10.1007/s00702-026-03184-2.

## Introduction

Multisensory integration (MSI)—the process of combining information from different sensory modalities—is essential for interpreting and responding to the environment efficiently. The brain determines what should be integrated based on several key principles. Temporal and spatial congruence play a fundamental role—stimuli occurring close in time and space are more likely to be integrated (Stein et al. 2020). Semantic congruence also influences MSI, as the brain favors the integration of stimuli that are meaningfully related, such as the sight of lips moving in sync with speech sounds (Letts et al. 2022). Additionally, the reliability of sensory cues is taken into account, with the brain weighting more reliable information more heavily in the integration process (Fetsch et al. 2012). However, several factors can disrupt or modify MSI. Environmental factors such as noise and distractions can impair MSI by affecting the clarity and salience of sensory stimuli (Foxe et al. 2020; Gibney et al. 2017). Additionally, cognitive factors like attention and memory deficits may also disrupt MSI (Macaluso et al. 2016; Quak et al. 2015). Sensory impairments, including hearing or vision loss, can further compromise MSI by reducing the availability or accuracy of sensory inputs (Freiherr et al. 2013; Peter et al. 2019). Moreover, ageing is another key factor that can affect MSI, as age-related changes in sensory processing and cognitive function impact this integration process (De Dieuleveult et al. 2017; Hernández et al. 2019; Setti et al. 2014).

In an aging world, studying diseases with rising prevalence, such as Parkinson’s Disease (PD), is crucial. PD is a chronic, progressive neurodegenerative disorder marked by motor symptoms—tremors, bradykinesia, rigidity, and postural instability—caused by dopaminergic neuron loss in the substantia nigra (Kalia & Lang 2015). Non-motor symptoms, including autonomic, cognitive, neurobehavioral, sleep, sensory, and perceptual impairments, also significantly affect function and quality of life (Chaudhuri & Schapira 2009). Sensory and perceptual impairments are particularly difficult to report because they are less visible to caregivers and clinicians, hard for patients to describe, and often not recognized as PD-related, despite their strong impact on daily life and well-being. MSI deficits, in particular, can subtly undermine balance, mobility, and the ability to respond to complex environments, without being captured in routine motor assessments (De Dieuleveult et al. 2017; Roytman et al. 2025). Furthermore, individuals with PD may lack insight into these deficits due to impaired self-awareness, compounding the challenge of recognition and reporting (Maier et al. 2023; Todorova et al. 2014).

Individuals with PD often experience cognitive impairment, ranging from mild deficits in perception and executive function to severe dementia (Caviness et al. 2007; Pagonabarraga & Kulisevsky 2012; Wallace et al. 2022). Since MSI is associated with cognitive ability (Hirst et al. 2022; Mahoney & Verghese 2020), impaired MSI in PD may exacerbate these deficits. Sensory abnormalities such as altered time perception (Artieda et al. 1992; Bernardinis et al. 2019; Lucas et al. 2013), visual and visuospatial difficulties (Alegret et al. 2001; José Luvizutto et al. 2020; Kemps et al. 2005; Lee et al. 1998), and reduced self- awareness (Mack et al. 2013; Maier & Prigatano 2017), may also reflect MSI dysfunction. MSI is critical for forming coherent perceptual experiences by integrating inputs across senses; when impaired, it can produce perceptual disturbances that limit accurate interaction with the environment. This raises the question of whether such disturbances stem from sensory abnormalities, MSI dysfunction, or both. MSI is also fundamental for motor control, integrating multimodal feedback to guide actions (Buchholz et al. 2012; Roy et al. 2021; Sober & Sabes 2003). Effective integration supports motor initiation, execution (Sober & Sabes 2003), mobility (Wu et al. 2015) and balance (Allison et al. 2006; Mahoney & Verghese 2020) Thus, MSI dysfunction may contribute to motor deficits in PD—including bradykinesia, gait disturbances, balance problems, and freezing of gait (FoG) (Olson et al. 2019) —ultimately worsening disability and overall impairment.

This review broadens the scope of previous studies on MSI in PD. While past research primarily focused on visual and vestibular integration related to gait and balance (Roytman et al. 2025), it systematically examines a wider array of sensory systems in PD, such as audio-visual and visuo-tactile processing. Its goal is to illuminate how the integration and coordination of multiple sensory inputs, crucial for cognitive function, perceptual accuracy, and postural stability, are affected in PD.

## Methods

### Population, phenomenon of interest, comparators, outcomes, study design

For this systematic review, a modified PICOS (Population, Phenomenon of Interest, Comparators, Outcomes, Study Design) framework was developed to guide the literature search and study selection, aligning with the review's objective to characterize MSI deficits in PD.

- *Population (P).* Individuals diagnosed with PD.
- *Phenomenon of Interest (I).* Multisensory Integration (MSI) and its various forms (e.g., audiovisual, visual-tactile, visual-vestibular integration, …), as assessed through experimental tasks.
- *Comparators (C).* Healthy control individuals, or comparisons within PD cohorts (e.g., different disease stages, on/off medication states, presence/absence of specific symptoms like freezing of gait or falls).
- *Outcomes (O).* The nature and extent of MSI deficits, their impact on motor functions (e.g., balance, postural control, movement accuracy) and non-motor functions (e.g., perceptual, cognitive), and the underlying neural mechanisms.
- *Study Design (S).* Original research studies (excluding reviews, conference abstracts, and intervention studies) were considered. No specific follow-up duration was required, as the review aimed to characterize observed deficits rather than assess longitudinal treatment effects.

This systematic review was conducted in accordance with the Preferred Reporting Items for Systematic Reviews and Meta-Analyses (PRISMA) guidelines (Moher et al. 2009). The initial literature search was performed between September 15–25, 2024, and was subsequently repeated on July 20, 2025, to capture the most recent publications. The protocol for this review has been registered in the PROSPERO database, an international prospective registry for systematic reviews, under the registration number CRD42024589213. A formal meta-analysis was not conducted due to the substantial heterogeneity observed across the included studies. Methodological variability, diverse experimental paradigms, and a wide range of outcome measures precluded a quantitative synthesis. This decision aligns with best practices for systematic reviews where statistical pooling of data is not appropriate due to significant differences in study designs.

### Study selection

**Fig. 1:**
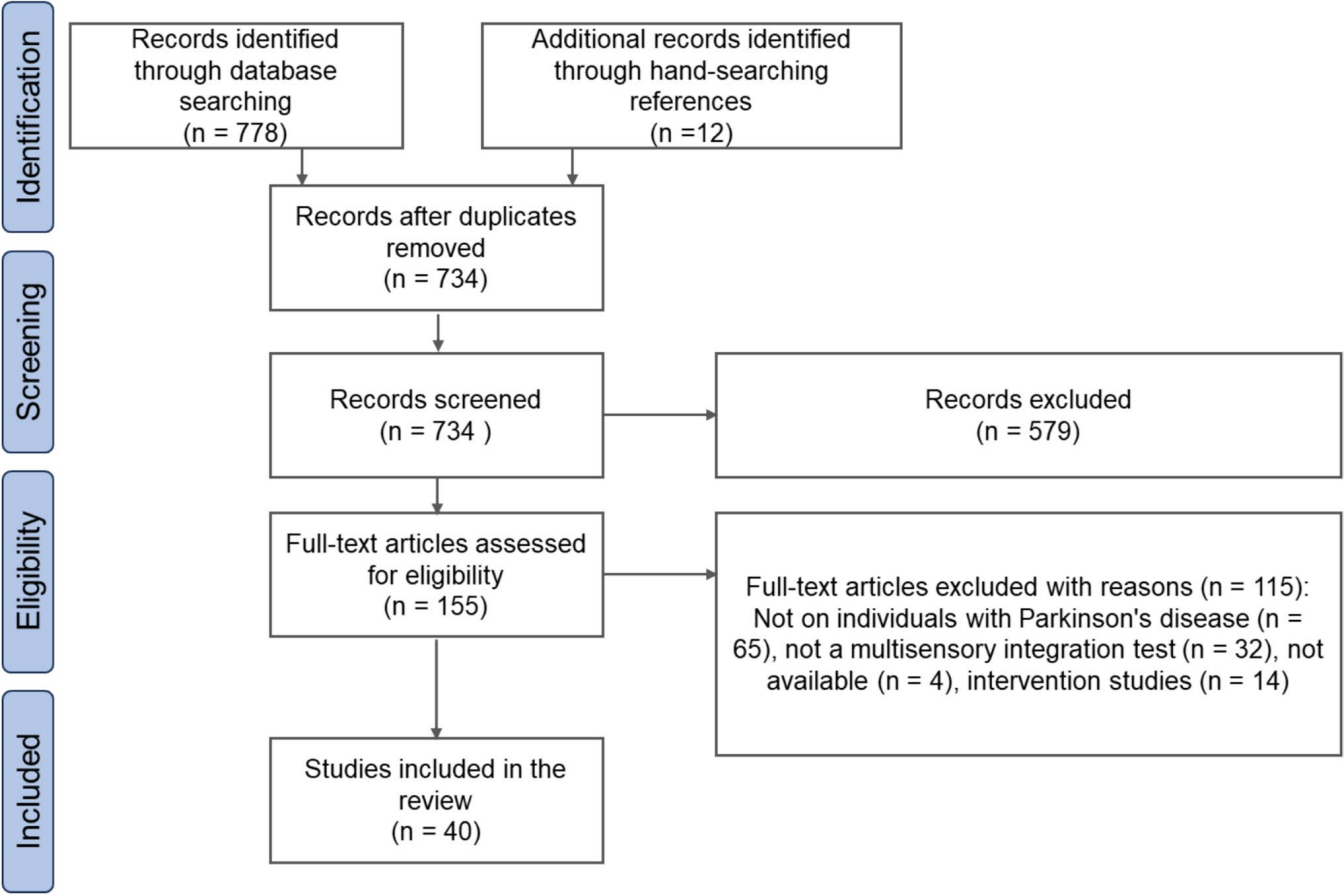
Flow of information through the different phases of the systematic review

The systematic review followed four PRISMA-based phases (Fig. 1): identification of records via database searches; screening, involving duplicate removal and application of selection criteria; eligibility, with full-text assessment against inclusion criteria; and inclusion of suitable articles. Title, abstract, and full-text screening were independently performed by two reviewers, with any discrepancies resolved through consensus. The first author made the final decision on the included studies.

The identification phase was performed in Scopus, the largest abstract and citation database of peer-reviewed literature (covering 100% of MEDLINE, EMBASE, and Compendex (Burnham 2006)), with records dating back to 1970. To improve comprehensiveness, manual searches were performed via backward and forward citation tracking of key articles (using tools such as Google Scholar), and AI-assisted tools—including Elicit, Research Rabbit, Connected Papers, and Semantic Scholar—were used to identify additional relevant studies through semantic similarity and citation network exploration. The search was limited to English-language articles to ensure accessibility, consistency, and critical evaluation, with no date restrictions.

### Terms

The literature search was conducted using a combination of search terms related to MSI and PD. Specifically, the search included the following terms: "sensory integration", "multisensory integration","crossmodal integration", "cross-modal integration", "intersensory integration", "multimodal integration", "crossmodal illusion", "multisensory illusion", "cross-modal illusion", "multisensory", "crossmodal", "cross-modal", "crossmodal sensory integration", "cross-modal sensory integration", and "multisensory interaction" in conjunction with "Parkinson's disease", "PD", "Parkinson", and "Parkinson disease" within the title, abstract, or keywords.

### Analysis of the results

The resulting studies were grouped by tested modality combinations. Microsoft Excel was used to organize search results, screen records, and manage inclusion/exclusion criteria, while R (Team, 2016) supported descriptive analysis and visualizations. Studies were described by key characteristics, and quality was assessed using the Newcastle–Ottawa Scale (NOS) (Wells et al. 2000) an eight-item tool for rating nonrandomized studies (case–control and cohort) across three sections: selection, comparability, and exposure.

## Results

### Quality assessment

All 40 studies were quality-rated using the NOS to assess risk of bias (Appendix 1, Supplementary Material). Most scored well (n = 37; Supplementary Table 1). As no meta-analysis was conducted, results from all studies are reported.

### Participants

Spanning over two decades of research (1999–2025), the literature demonstrates a sustained interest in this topic. Notably, 25 of the 40 included studies (62.5%) were published in the last decade (2016–2025), with 9 of these (22.5%) published between 2021 and 2025. This confirms that multisensory dysfunction remains an active and evolving area of PD research. Geographically, this research spans a global demographic. The majority of studies were conducted in the USA (n = 15), followed by Australia (n = 4), Japan (n = 3), Canada (n = 3), Italy (n = 3), and one each from Ireland, Iran, UK, Germany, Spain, Belgium, France, South Korea, India, Israel, China, and Brazil. In total, 2,070 participants were analyzed (1,150 PD patients, 920 healthy controls; Table 1). Four studies lacked a control group (Freeman et al. 2018; Gera et al. 2016; Harro et al. 2016; Hwang et al. 2016). Most used healthy age-matched controls (HAMC), some also included young healthy groups (Bek et al. 2022; Konczak et al. 2012; Nallegowda et al. 2004; Sabaté et al. 2008; Shoji et al. 2024), while one study used non-age-matched controls (Scarpina et al. 2019a). In (Hwang et al. 2016), a separate study with the same task used a young healthy group as controls. PD group sizes ranged from 8 (Hwang et al. 2016) to 124 (Müller et al. 2013) (mean = 28.8), and HC group sizes from 9 (Feller et al. 2019) to 65 (Bek et al. 2022) (mean = 25.6). Additional subgroups included on/off medication (Barnett-Cowan et al. 2010; Ding et al. 2017, 2018; Feller et al. 2019; Nallegowda et al. 2004; Sabaté et al. 2008; Waldmann et al. 2020; Yakubovich et al. 2020), FoG vs. non-FoG (Fearon et al. 2015; Huh et al. 2016), fallers vs. non-fallers (DiFrancisco-Donoghue et al. 2015), people with Alzheimer’s disease (AD) (Chong et al. 1999), and individuals with nonthalamic subcortical or cortical lesions in addition to PD (Copland et al. 2000).

**Table 1:** Summary of studies assessing MSI in PD, including participant characteristics, cognitive status, sensory modalities tested, and key findings

No	Authors (Year)	Sample size	H&Y stage (UPDRS if missing)	Medication status	Cognitive assessment	MSI modalities tested	Key findings
1	Rostami et al. (2024)	15 PD, 15 HAMC	1–3	Off-medication (≥ 12 h)	MoCA (no difference)	Visual, Auditory	Wider TBW, visual-first harder
2	Ren et al. (2018)	30 PD, 36 HAMC	2.4 (SD = 0.1)	On-medication	MoCA (no difference)	Visual, Auditory	Abnormal audiovisual, delayed RT
3	Harrington et al. (2014)	27 PD, 24 HAMC	60% stage 2); remainder stage 3	Off-medication	MMSE > 25	Visual, Auditory	Abnormal intersensory timing); altered connectivity
4	Zhou et al. (2025)	27 PD, 22 HAMC	1.85 (SD = 0.58)	On-medication	MMSE (no difference)	Visual, Auditory	Abnormal audiovisual conflict during perception not response
5	Fearon et al. (2015)	39 PD, 17 HAMC	2.5 (SD = 1–4)	On-medication	MoCA (no difference)	Visual, Auditory	Slower RT, weak multisensory gain
6	Copland et al. (2000)	10 PD, 10 HAMC 10 non-thalamic subcortical lesions; 10 cortical lesions (results not shown)	2.75 (SD = 0.825)	On-medication	MDRS used to exclude dementia	Visual, Auditory	Impaired auditory-visual semantic integration
7	Waldmann et al. (2020)	42 PD, 48 HAMC (17 PD tested OFF)	UPDRS III ON: 25 (17–31); OFF: 41 (29.5–45)	Both ON and OFF	PANDA	Visual-Tactile, Proprioception	Higher RHI, med state no effect
8	Ding et al. (2018)	24 PD, 21 HAMC	II–IV	Both ON and OFF	MoCA (no difference)	Visual-Tactile, Proprioception	RHI improved with STN-DBS
9	Ding et al. (2017)	21 PD, 21 HAMC	I–III; MDS-UPDRS III ON: 13.5; OFF: 26.5	Both ON and OFF	MoCA (no difference)	Visual-Tactile, Proprioception	Greater RHI ON medication
10	Rabin et al. (2010)	9 PD, 9 HAMC	Mild (UPDRS 18–31)	Off-medication (≥ 12 h)	Modified MMSE > 40/57	Tactile, Proprioception	Tactile improves proprioceptive deficits
11	Shoji et al. (2024)	32 PD, 32 HAMC, 38 younger HC	UPDRS-III 23.8 (12.1)	None reported	MMSE	Visual, Respiratory (interoceptive)	Aging—not PD—reduces body ownership recalibration via vision-respiratory integration
12	Konczak et al. (2012)	12 PD, 12 HAMC, 12 younger HC	2.42 (SD = 0.69)	On-medication	MMSE > 26	Proprioceptive, Tactile	PD disrupts haptic precision integration
13	Freeman et al. (2018)	26 PD HAMC	2–3	On-medication	None reported	Visual, Vestibular, Somatosensory	Quantifies multisensory balance control
14	Tran et al. (2023)	13 PD, 13 HAMC	1–3	On-medication	None reported	Visual, Vestibular	Visual-vestibular gait preserved
15	Bohnen et al. (2022a, b)	106 PD, 29 HAMC	2.4 ± 0.6 (1–4)	On-medication	MoCA	Visual, Vestibular, Somatosensory	Vestibular loss linked to imbalance
16	Bohnen et al. (2022a, b)	92 PD, 21 HAMC	Mean 2.5 ± 0	Off-medication	MoCA	Vision, Proprioception, Vestibular	Cholinergic loss linked to FoG
17	Hawkins et al. (2021)	40 PD, 40 HAMC	78% in H&Y I–II	On-medication	None reported	Vestibulo-visual	Higher visual dependence, low threshold
18	Müller et al. (2013)	124 PD, 25 HAMC	Mean 2.4 ± 0.5	Off-medication	MoCA	Vestibular, Somatosensory	Thalamic cholinergic loss affects sway
19	Barnett-Cowan et al. (2010)	12 PD, 13 HAMC	Mean 2.5 (1–2.5)	9 tested both ON and OFF	MMSE > 26	Visual, Vestibular, Body cues	Visual dep. ↑ with meds, Worse on-medication
20	Hwang et al. (2016)	8 PD, separate young HC group	Mean 1.75 ± 0.75	On-medication	MMSE > 25	Visual, Vestibular, Proprioception	Impairment in cross-modal sensory reweighting
21	Yakubovich et al. (2020)	19 PD (tested ON/OFF), 23 HAMC, 20 younger HC	Early-stage	Both ON and OFF	MoCA (no difference)	Visual, Vestibular	Visual motion impaired; vestibular spared
22	Huh et al. (2016)	47 PD (25 with FoG, 22 without), 26 HAMC	2–3 (mean 2.5)	Off-medication	MoCA (no difference)	Visual, Vestibular, Somatosensory	Vestibular/somatosensory loss → FoG
23	Nallegowda et al. (2004)	30 PD, 30 HAMC	Mean 2.7	Both ON and OFF	None reported	Vision, Proprioception	Med improves gait, strength, SOT
24	Harro et al. (2016)	42 PD	Mean 2.3 (range I–IV)	On-medication	MoCA (cutoff 21/30)	Multiple sensory modalities (balance, postural control)	SOT/LOS reliable for PD balance
25	Gera et al. (2016)	45 PD	Mean 2.4 ± 0.65	On-medication	No deficits reported	Visual, Vestibular	Mini-BEST more sensitive than SOT
26	DiFrancisco-Donoghue et al. (2015)	19 PD (10 non-fallers, 9 fallers), 10 HAMC	Mild to moderate (UPDRS-based)	On-medication	None reported	Visual, Vestibular	Learning effect in fallers
27	Chong et al. (1999)	15 PD, 11 AD, 17 HAMC	III–IV	On-medication	Not for PD	Visual, Somatosensory, Vestibular	PD = general balance deficits
28	Colnat-Coulbois et al. (2011)	24 PD, 48 HAMC	Late-stage, H&Y IV	On-medication	None reported	Visual, Vestibular, Proprioception	Hip-dominant strategy; poor precision
29	Sadeghi et al. (2025)	10 PD, 11 HAMC	Median 2 [1.5–2.5]	On-medication	MoCA > 20	Visual, Vestibular, Somatosensory	↑ cortical activity = MSI issue
30	Feller et al. (2019	10 PD, 9 HAMC	2 [1–3]	Both ON and OFF	None reported	Vision, Proprioception	Proprio threshold high; meds no effect
31	José Luvizutto et al. (2020)	13 PD, 14 HAMC	< 3	On-medication	Excluded if cognitive dysfunction	Visual, Vestibular, Somatosensory	Altered haptic verticality perception
32	Harro et al. (2018)	42 PD, 55 HAMC	Not reported	On-medication	MoCA (cutoff 21/30)	Visual, Vestibular	Vestibular motion perception worse
33	Vervoort et al. (2013)	19 PD (9 Freezers, 10 Non-freezers), 10 HAMC	Between II and IV during the “on” state	On-medication	MMSE > 24	Visual, Vestibular, Proprioceptive	Freezers show voluntary shift deficits
34	Adamovich et al. (2001)	9 PD, 9 HAMC	II–III	Off-medication	None reported	Visual, Proprioceptive	Poor visual-proprio integration; ↑ variability
35	Brown et al. (2006)	12 PD, 12 HAMC	UPDRS 19.42 (SD = 9.39)	On-medication	None reported	Visual, Proprioceptive, Vestibular	Poor sensory reorganization post-vision loss
36	Bek et al. (2022)	46 PD, 30 younger HC, 35 HAMC	I–III	On-medication	ACE (Addenbrooke’s Cognitive Examination)	Visual, Proprioceptive, Kinesthetic	Impaired imagery; MSI affects rehab
37	Scarpina et al. (2019a, b)	20 PD (10 left-, 10 right-lateralized), 20 HAMC	Mild to moderate (UPDRS)	On-medication	MMSE	Visual, Proprioceptive, Kinesthetic	Impaired left-hand imagery
38	Scarpina et al. (2019a, b)	14 PD, 18 HC (not age-matched)	2.29 (SD = 0.76)	On-medication	MMSE > 24	Visual, Proprioceptive, Kinesthetic, Tactile	Tool embodiment impaired, body plasticity reduced
39	Sabaté et al. (2008)	10 PD, 10 HAMC	1.8 (SD = 2.2)	Both ON and OFF	No deficits reported	Auditory, Kinesthetic	No sensory reweighting
40	Honma et al. (2018)	17 PD, 18 HAMC	2.73 (SD = 0.96)	On-medication	MMSE > 25 MoCA > 25	Visual, Olfaction	Impaired visual modulation of olfactory perception

*PD* Parkinson’s disease, *MSI* Multisensory integration, *HAMC* Healthy age-matched controls, *HC* Healthy controls (not age-matched), *H&Y* Hoehn & Yahr stage, *UPDRS* Unified Parkinson’s Disease Rating Scale, *MDS-UPDRS* Movement Disorder Society-Unified Parkinson's Disease Rating Scale, *MMSE* Mini-Mental State Examination, *MoCA* Montreal Cognitive Assessment, *TBW* Temporal binding window, *SOT* Sensory Organization Test, *STN-DBS* Subthalamic Nucleus Deep Brain Stimulation, *FoG* freezing of gait, *RHI* Rubber Hand Illusion, *PANDA* Parkinson Neuropsychometric Dementia Assessment

*Medication status.* Across the included studies, seven tested participants in the OFF-medication state (typically after 12-h withdrawal) (Adamovich et al. 2001; Bohnen et al. 2022a; Harrington et al. 2014; Huh et al. 2016; Müller et al. 2013; Rabin et al. 2010; Rostami et al. 2024), twenty-four in the ON-medication state using their regular dopaminergic regimen (Bek et al. 2022; Bohnen et al. 2022a; Brown et al. 2006; Chong et al. 1999; Colnat-Coulbois et al. 2011; Copland et al. 2000; DiFrancisco-Donoghue et al. 2015; Fearon et al. 2015; Freeman et al. 2018; Gera et al. 2016; Harro et al. 2016, 2018; Hawkins et al. 2021; Hwang et al. 2016; José Luvizutto et al. 2020; Konczak et al. 2012; Readman et al. 2021; Ren et al. 2018; Sadeghi et al. 2025; Scarpina et al. 2019a, b; Tran et al. 2023; Vervoort et al. 2013; Zhou et al. 2025), and eight in both states to compare medication effects (Barnett-Cowan et al. 2010; Ding et al. 2017, 2018; Feller et al. 2019; Nallegowda et al. 2004; Sabaté et al. 2008; Waldmann et al. 2020; Yakubovich et al. 2020). One study did not specify the medication status (Shoji et al. 2024).

*Cognition.* Most studies controlled for cognitive function using the Montreal Cognitive Assessment (MoCA) or Mini-Mental State Examination (MMSE), with no significant baseline differences between PD and controls reported in many cases (Bohnen et al. 2022a, b; Ding et al. 2017, 2018; Fearon et al. 2015; Harro et al. 2016, 2018; Huh et al. 2016; Müller et al. 2013; Ren et al. 2018; Rostami et al. 2024; Sadeghi et al. 2025; Tran et al. 2023; Yakubovich et al. 2020; Zhou et al. 2025). Exclusion criteria varied, with MoCA cut-offs at 21 or 25 (Harro et al. 2016; Honma et al. 2018; Sadeghi et al. 2025), MMSE cut-offs at 24–26 (Barnett-Cowan et al. 2010; DiFrancisco-Donoghue et al. 2015; Honma et al. 2018; Hwang et al. 2016; Konczak et al. 2012; Scarpina et al. 2019a; Vervoort et al. 2013), and other measures such as the Modified MMSE (> 40/57) (Rabin et al. 2010), PANDA (Waldmann et al. 2020), and Addenbrooke’s Cognitive Examination (Bek et al. 2022). Some studies excluded participants with cognitive dysfunction altogether (Adamovich et al. 2001; José Luvizutto et al. 2020) to ensure sample homogeneity. 

*PD stage.* Most studies involved Hoehn and Yahr (H&Y) stages I–III (Adamovich et al. 2001; Bek et al. 2022; Hawkins et al. 2021; Konczak et al. 2012; Rostami et al. 2024; Tran et al. 2023; Zhou et al. 2025) or mean stages around 2.4–2.7 (Bohnen et al. 2022b; Honma et al. 2018; Huh et al. 2016; Müller et al. 2013; Ren et al. 2018), indicating mild to moderate PD. A few included advanced cases (stages III–IV) (Chong et al. 1999; Colnat-Coulbois et al. 2011). Severity was further characterized using UPDRS or MDS-UPDRS Part III consistently supporting the classification of participants within mild to moderate stages of PD. These assessments were frequently conducted in both ON and OFF medication states to capture the effects of dopaminergic therapy. Many studies (e.g., Barnett-Cowan et al. 2010; Ding et al. 2017, 2018; Harrington et al. 2014; Rabin et al. 2010; Waldmann et al. 2020; Yakubovich et al. 2020) found higher motor scores in the OFF state and improved performance in the ON state, indicating typical medication responsiveness. Some assessed only the ON state e.g., (Harro et al. 2016; Konczak et al. 2012; Vervoort et al. 2013), usually at peak effect, while others tested only the OFF state (e.g., Huh et al. 2016; Nallegowda et al. 2004). H&Y staging was reported in the ON state (e.g., Colnat-Coulbois et al. 2011), the OFF state (e.g., Nallegowda et al. 2004), or in both (e.g., Harrington et al. 2014), with OFF-state ratings often preferred as a better reflection of baseline motor severity. Some studies (e.g., DiFrancisco-Donoghue et al. 2015) excluded participants with major motor fluctuations or advanced dyskinesia to ensure stable assessments. Several studies did not specify the state of medication when testing for PD stage, which reduced comparability.

*MSI assessment tasks across modalities.* MSI paradigms typically involve the presentation of congruent or incongruent stimuli across sensory modalities, with manipulating of spatial/temporal alignment or the introduction of conflicts to study effects on perception and motor responses. Research on PD and MSI has explored two main areas: postural control, which relates to motor function and balance (for review, see [Roytman et al. 2025]), and perceptual integration, which deals with how the brain processes sensory information. Outcome measures include reaction time, perceptual accuracy, postural control, and task performance. Studies can be grouped by task type: many employed the Sensory Organization Test (SOT) to assess balance and sensory weighting (Bohnen et al. 2022b; Chong et al. 1999; Colnat-Coulbois et al. 2011; DiFrancisco-Donoghue et al. 2015; Freeman et al. 2018; Gera et al. 2016; Harro et al. 2018; Huh et al. 2016; Nallegowda et al. 2004; Sadeghi et al. 2025; Vervoort et al. 2013). The SOT evaluates how individuals maintain postural stability under conditions in which visual, vestibular, and somatosensory cues are selectively altered or removed. Studies assessing gait and balance control through dynamic or immersive platforms include those by (Brown et al. 2006; Hawkins et al. 2021; Hwang et al. 2016; Tran et al. 2023). Additional motor tasks included arm-matching and grasping task (Adamovich et al. 2001; Rabin et al. 2010), active vs. passive haptic exploration (Konczak et al. 2012), and tool-use adaptation involving pointing and tactile estimation (Scarpina et al. 2019a). In perceptual integration, the Rubber Hand Illusion (RHI) was used to study body ownership and proprioceptive recalibration (Ding et al. 2017, 2018; Shoji et al. 2024; Waldmann et al. 2020). Audiovisual paradigms included the Sound-induced Flash Illusion task (Rostami et al. 2024), which tests how a single visual flash paired with sound can create the illusion of multiple flashes. Audiovisual matching tasks (Ren et al. 2018; Zhou et al. 2025) and semantic integration paradigms (Copland et al. 2000) were used, with the latter showing effects of cross-modal congruency on lexical decisions. Other modalities were explored, such as olfactory-visual integration (Honma et al. 2018), which assessed how odor judgments were modulated by semantically congruent or incongruent images. Finally, motor imagery and internal representation tasks (Bek et al. 2022; Scarpina et al. 2019b) were employed to assess body schema and movement simulation.

### Findings on MSI by modality

#### Visual-vestibular integration

Individuals with PD rely more heavily on visual cues for balance (Brown et al. 2006; Hawkins et al. 2021), a condition known as visual dependency. While their gait may be comparable to controls with visual input, they fail to adjust their gait as cautiously under vestibular stimulation (Tran et al. 2023). They also show impaired sensory reweighting (Sadeghi et al. 2025), impaired visual self-motion perception despite intact vestibular function (Yakubovich et al. 2020) and increased visual and gravitational dependence for perceptual uprightness (Barnett-Cowan et al. 2010). Cholinergic dysfunction, specifically in the thalamus, is a key predictor of postural instability and falls, even more so than dopaminergic loss (Bohnen et al. 2022a, b; Müller et al. 2013). The SOT has been instrumental in characterizing these balance issues. PD patients show a reduced ability to integrate visual, vestibular, and proprioceptive cues, with no reweighting of sensory inputs in response to perturbations (Hwang et al. 2016). A higher reliance on visual information is linked to greater FoG severity (Huh et al. 2016). While some studies noted that voluntary weight shifting is more impaired in PD freezers than in non-freezers (Vervoort et al. 2013), others highlight that PD patients have a higher threshold for proprioceptive signal detection, which can hinder balance corrections (Feller et al. 2019). Muscle weakness and sensory integration impairments also contribute to postural instability, with some improvement seen under medication (Nallegowda et al. 2004). Clinically, balance assessments such as the SOT, Limits of Stability, and Motor Control Test are reliable for monitoring disease severity and fall risk (DiFrancisco-Donoghue et al. 2015; Harro et al. 2016), while the Mini-BESTest is more sensitive for detecting deficits (Gera et al. 2016). Comparisons also show broader balance impairments in PD relative to selective sensory integration deficits in Alzheimer’s disease (Chong et al. 1999). Reduced postural precision, increased sway, and heightened cortical involvement during balance tasks further underscore impaired sensory integration in PD (Colnat-Coulbois et al. 2011; Sadeghi et al. 2025).

#### Audiovisual integration

Audiovisual processing deficits in PD are highlighted in several studies. Patients exhibit a significantly wider temporal binding window (TBW) (Rostami et al. 2024), indicating difficulty in synchronizing auditory and visual inputs—a fundamental disruption in MSI. This alteration in temporal integration is so pronounced that some studies report a complete absence of audiovisual integration in response to peripheral stimuli in PD patients, regardless of cognitive or sleep issues (Ren et al. 2018). Fearon et al. (2015) found that PD patients retain multisensory facilitation, but at a reduced level compared to controls. Auditory stimuli were processed faster than visual ones, with this temporal MSI shift linked to disease progression, FOG, bradykinesia, and other gait disturbances. Furthermore, PD patients struggle with cross-modal contextual integration in language processing (Copland et al. 2000), suggesting a breakdown in frontal-striatal mechanisms. Neuroimaging studies have linked impaired temporal processing of audiovisual signals to abnormal functional connectivity within cortico-thalamo-basal ganglia circuits (Harrington et al. 2014). The observation that models based on perceptual conflict classify PD more accurately than those based on motor responses underscores that these perceptual impairments are not merely secondary to motor symptoms (Zhou et al. 2025).

#### Visual-tactile and proprioceptive integration

The integration of visual and tactile information is significantly impaired, leading to a disrupted sense of body ownership. PD patients show increased susceptibility to the RHI (Waldmann et al. 2020), even under conditions that do not induce it in healthy individuals. This deficit is not normalized by dopaminergic medication (Ding et al. 2017), but deep brain stimulation (DBS) of the subthalamic nucleus (STN) can partially restore this function (Ding et al. 2018).

#### Other modalities

*Visuo-Olfactory.* PD patients show impaired visuo-olfaction integration, where visual cues fail to influence odor judgments (Honma et al. 2018). This is linked to reduced dopamine transporters (DaT) in the posterior putamen, suggesting a potential early diagnostic marker (Honma et al. 2018).

*Tactile-proprioceptive.* While a study suggests the mechanism for prioritizing tactile and proprioceptive feedback is intact (Rabin et al. 2010), another finds that PD significantly reduces haptic sensitivity and acuity (Konczak et al. 2012), indicating that motor issues might stem from underlying somatosensory dysfunction.

*Visual-proprioceptive.* PD patients struggle to integrate proprioceptive and visual feedback for accurate movements (Adamovich et al. 2001), and show impaired perception of verticality.

*Visual, kinesthetic, tactile, and proprioceptive.* PD patients show deficits in motor imagery and body schema representation (Bek et al. 2022; Scarpina et al. 2019b), They fail to update their body representation during tool use, a process that healthy individuals demonstrate (Scarpina et al. 2019a).

*Auditory-kinesthetic.* The integration of auditory and kinesthetic stimuli is altered in a task-dependent manner in PD, affecting motion control (Sabaté et al. 2008).

*Visuo-interoceptive.* In contrast to other deficits, recalibration of body ownership in a vision-respiratory RHI paradigm appears to be influenced by aging rather than PD itself (Shoji et al. 2024).

### Neuroimaging findings

While behavioral studies dominate MSI research in PD, neuroimaging has provided key insights into underlying mechanisms. Deficits in vestibular function and thalamic cholinergic loss are significant predictors of imbalance, as shown by dynamic posturography and PET (Bohnen et al. 2022b). Cholinergic deficits in regions such as the medial geniculate nucleus and para-hippocampal gyrus contribute to vestibular sensory conflicts and motor symptoms (Bohnen et al. 2022a). EEG during virtual reality tasks indicates altered cortical activity and impaired sensory reweighting for postural control (Sadeghi et al. 2025). fMRI shows that subthalamic nucleus deep brain stimulation (STN-DBS) improves MSI, with altered basal ganglia and anterior insula activity (Ding et al. 2018). Reduced thalamic cholinergic innervation, rather than cortical or striatal deficits, correlates with increased postural sway, underscoring the thalamus’s role (Müller et al. 2013). Abnormal cortico-thalamus-basal ganglia connectivity and enhanced cerebellar connectivity are linked to impaired audiovisual temporal processing (Harrington et al. 2014). Structural MRI further reveals that abnormal audiovisual conflict in PD occurs specifically during perception, correlating with reduced cortical thickness in the left middle frontal gyrus (Zhou et al. 2025). Beyond visual-vestibular and auditory domains, PD patients also show impaired visual-olfactory integration, with neuroimaging linking this to reduced dopamine transporter availability in the posterior putamen (Honma et al. 2018).

### Medication findings

Dopaminergic medication yielded varied effects on sensory and multisensory processing in PD. It was observed to influence sensory re-weighting, including impaired proprioceptive reweighting in balance tasks which consistently remained unaffected by medication (Feller et al. 2019). Medication was associated with improvements in muscle strength and gait while leaving proprioception intact (Nallegowda et al. 2004). Increased susceptibility to the RHI was also reported under medication (Ding et al. 2017). In contrast, several paradigms found no ON–OFF differences, suggesting that some deficits, such as temporal binding impairments or elevated internal noise, may not depend on dopaminergic modulation (Waldmann et al. 2020). In addition, patients demonstrated an over-weighting of degraded visual cues (Yakubovich et al. 2020), indicating that certain sensory pathways may be relatively resistant to dopaminergic influence.

### Summary of findings

**Fig. 2:**
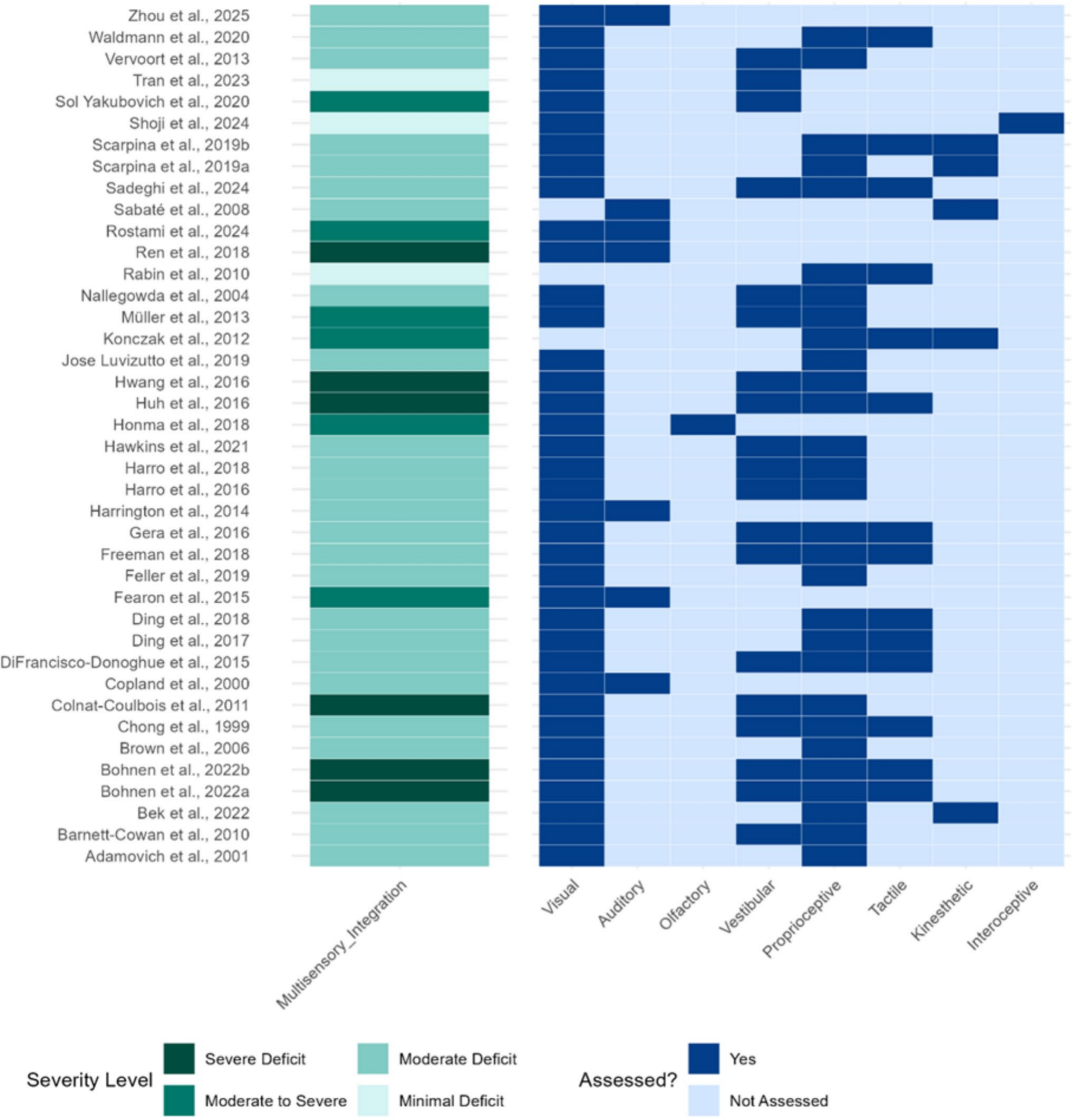
Severity of Multisensory Integration and Sensory Modality Assessment Across Studies. Each row in this table corresponds to a specific study. The "Multisensory_Integration" column indicates the overall severity of MSI impact observed in that study, color-coded from light teal (Minimal/Mild deficits) to dark teal (Severe deficits). The other columns (e.g., Visual, Auditory, Vestibular) show whether that specific sensory modality was assessed in the study: dark blue signifies "Yes" (it was studied), light blue indicates "No" (it was not studied)

Figure 2 presents a heatmap summarizing the severity of MSI impairments across the included studies. Each row represents a study, and columns correspond to sensory modalities—Visual, Auditory, Vestibular, Proprioceptive, Tactile, Kinesthetic, Olfactory, and Interoceptive—along with overall MSI outcomes. The leftmost column indicates whether MSI deficits were associated with PD, while the remaining columns show whether sensory deficits were examined within each study. MSI is widely impacted, with deficits ranging from *Minimal* to *Severe* across different sensory modalities. Visual, Proprioceptive, and Vestibular integration are the most frequently studied and often show significant impairments (commonly categorized as *Moderate to Severe* due to altered temporal reweighting and postural instability). Auditory, Tactile, and Kinesthetic integration are also affected, albeit with more variability. Some specific combinations, such as auditory–visual and olfactory–visual integration, demonstrate consistent and often severe breakdowns, whereas others—like certain proprioceptive–tactile interactions or aspects of visual–vestibular integration in mild PD—show minimal direct impact. Overall, these findings highlight that PD significantly compromises the brain’s ability to integrate multisensory information, thereby contributing to both motor dysfunction and perceptual difficulties.

To further synthesize these findings and provide a clearer structured comparison, Table 2 outlines the common experimental paradigms, primary deficits observed, and their cascading impacts on motor and cognitive function across modalities. Together, these findings highlight that PD significantly compromises the brain’s ability to integrate multisensory information, thereby contributing to both motor dysfunction and perceptual difficulties.

**Table 2:** Structured comparison of MSI deficits and functional impacts in PD

Sensory modality	Common experimental paradigms	Primary deficits observed	Impact on motor/cognitive function
Visual-Vestibular	SOT, Dynamic/immersive VR platforms	Impaired sensory reweighting; visual dependency; impaired visual self-motion perception	Increased postural instability; higher risk of falls; exacerbation of FOG; abnormal gait adjustments
Audiovisual	Sound-induced Flash Illusion, Audiovisual matching, Semantic integration	Widened TBW; reduced or absent multisensory facilitation	Associated with bradykinesia and gait disturbances; impaired cross-modal language processing
Visual-Tactile & Proprioceptive	RHI, Arm-matching, Tool-use adaptation	Disrupted sense of body ownership; altered proprioceptive recalibration; reduced haptic sensitivity	Impaired accurate movement execution; deficits in motor imagery and updating body schema during tool use
Visuo-Olfactory	Odor judgments with congruent/incongruent images	Visual cues fail to appropriately influence odor judgments	Correlates with reduced dopamine transporters; potential early cognitive/diagnostic marker
Tactile-Proprioceptive	Active vs. passive haptic exploration	Reduced haptic sensitivity and acuity	Contributes to underlying motor execution issues and somatosensory dysfunction
Visual-Proprioceptive & Kinesthetic	Arm-matching, grasping tasks, motor imagery	Impaired perception of verticality; deficits in internal body representation	Impaired movement accuracy, motor imagery, and updating body schema during tool use
Auditory-Kinesthetic	Task-dependent motion tracking	Altered temporal integration of sound and physical movement	Disruptions in precise motion control
Visuo-Interoceptive	Vision-respiratory RHI	No PD-specific deficit observed (effects driven by aging)	Body ownership recalibration via interoception remains relatively intact

*PD* Parkinson’s disease, *MSI* Multisensory integration, *FOG* Freezing of Gait, *TBW* Widened temporal binding window, *RHI* Rubber Hand Illusion, *SOT* Sensory organization test

## Discussion

Research on PD has moved beyond just motor symptoms, with a growing body of evidence highlighting significant deficits in MSI. These impairments affect both postural control and perceptual integration, often mirroring and exacerbating age-related declines.

### MSI deficits in PD and their parallels in older adults

MSI deficits in individuals with PD are closely linked to those observed in older adults, as both groups experience declines in the ability to integrate multisensory information. In healthy aging, MSI naturally deteriorates, leading to slower processing and less accurate perceptions (Hernández et al. 2019; Mahoney et al. 2011), which is associated with an increased risk of falls and balance difficulties (Mahoney et al. 2019; Setti et al. 2011; Zhang et al. 2020). PD further exacerbates these issues. For example, the greater reliance on vision for posture in PD (Barnett-Cowan et al. 2010) mirrors the compensatory reliance on vision seen in older adults as their vestibular and proprioceptive systems decline (Jeka et al. 2010). Similarly, the difficulty in integrating auditory and kinesthetic cues in motor control (Sabaté et al. 2008) reflects the broader coordination challenges older adults face. While both older adults (Setti et al. 2011) and individuals with PD (Rostami et al. 2024) exhibit enlarged TBW, the impairment is more severe in PD. This suggests that although aging contributes to MSI decline, the neurodegenerative processes in PD impose an additional and significant burden on integrative abilities, resulting in more pronounced functional impairments and a higher fall risk compared to healthy older adults.

### Balance and postural control

Individuals with PD have significant difficulties with visual-vestibular integration during gait, a major factor in their balance issues and increased fall risk (Bohnen et al. 2022b; Tran et al. 2023). The severity of these deficits worsens with disease progression, with advanced PD showing more severe vestibular impairments compared to visual ones (Hwang et al. 2016). Vestibular deficits in PD worsen fall risk by impairing both static and dynamic balance (Rinalduzzi et al. 2015). Evidence indicates that targeted interventions addressing vestibular dysfunction may improve stability and reduce falls, underscoring the need for further research.

Cholinergic systems also play a central role. Impaired thalamic cholinergic innervation in PD disrupts the integration of somatosensory and vestibular cues during postural challenges, compromising the ability to resolve conflicting inputs and adapt to perturbations (Müller et al. 2013). Reduced cholinergic function not only increases fall risk in PD (Bohnen et al. 2022a) but also interacts with aging-related declines in cholinergic processing (Montero-Odasso et al. 2009), further undermining balance in older adults. These findings underscore the potential of targeting cholinergic pathways in fall-prevention strategies. Impaired postural stability in PD reflects not only cholinergic factors but also basal ganglia dysfunction, as patients fail to reintegrate sensory inputs after visual removal, leading to persistent sway (Brown et al. 2006), alongside broader sensorimotor integration deficits such as degraded haptic sensitivity and impaired proprioceptive–tactile fusion (Konczak et al. 2012) and reduced adaptability of body schema following tool use (Scarpina et al. 2019a). Despite these challenges, some somatosensory mechanisms, such as proprioceptive-tactile integration, often remain intact (Rabin et al. 2010), offering a potential compensatory pathway to support balance (Roytman et al. 2025). Advanced tools like VR-based tasks and portable monitors are essential for assessing these complex deficits (Freeman et al. 2018; Hawkins et al. 2021), enabling the detection of sensory-motor dysfunctions such as reduced adaptability in body schema following tool use (Scarpina et al. 2019b) and supporting targeted, personalized strategies to enhance balance.

### Perceptual integration

PD also involves significant perceptual integration abnormalities, which can serve as early indicators of the disease.

#### Body ownership

Altered body ownership perceptions, as evidenced by the RHI studies (Ding et al. 2017, 2018; Waldmann et al. 2020), underscore significant MSI issues in PD. These disruptions in integrating sensory inputs have tangible implications for daily functioning and self-awareness by affecting tasks that require precise motor control and spatial awareness (Ricciardi et al. 2016). Research indicates that the experience of body ownership can activate the motor system even without physical movement (Ehrsson et al. 2004; Tsakiris 2010). Further supporting this idea, body ownership illusions can activate motor areas as if the perceived body were moving (Petkova & Ehrsson 2008), and even the illusion of owning an "invisible hand" can engage the motor system without overt movement (Guterstam et al. 2013). VR-based body ownership experiences could therefore offer a promising avenue to enhance motor recovery patients (Kokkinara et al. 2016; Tambone et al. 2021). However, a nuanced relationship exists between PD, aging, and specific aspects of body ownership recalibration; one study found that aging, not PD, primarily affects recalibration linked to interoceptive cues (Shoji et al. 2024).

#### Audiovisual integration and timing deficits

Studies highlight audiovisual integration and intersensory timing deficits (Fearon et al. 2015; Ren et al. 2018; Rostami et al. 2024), serving as early disease indicators and a core feature of PD's MSI impairments. These timing abnormalities often manifest as an exaggerated underestimation of durations (Harrington et al. 2014) or a widened TBW (Rostami et al. 2024). Cognitive symptoms, particularly frontal cortex-linked executive dysfunction, further influence these deficits (Parker et al. 2013). Recent research indicates abnormal audiovisual conflict is a perceptual issue, manifesting at the level of perception rather than response, with cortical thinning in the left middle frontal gyrus correlating with this interference (Zhou et al. 2025). Beyond basic timing, language studies reveal PD patients struggle with maintaining context-based word meaning, reflecting frontal-striatal semantic integration deficits (Copland et al. 2000).

### Impact of dopaminergic treatment

Dopaminergic treatment in PD has complex and sometimes contradictory effects on MSI. While dopaminergic drugs effectively alleviate motor symptoms (Rascol et al. 2003; Wei & Shetty 2021), they may simultaneously exacerbate or reveal perceptual instabilities. Dopaminergic treatment effects on MSI are varied: it alters sensory re-weighting, including impaired proprioceptive reweighting in balance tasks that remain unaffected by medication (Feller et al. 2019), and improve muscle strength and gait without impairing proprioception (Nallegowda et al. 2004). While it can increase RHI susceptibility (Ding et al. 2017), some deficits, like temporal binding impairments or increased internal noise, show no ON–OFF differences, suggesting dopamine independence (Waldmann et al. 2020). Furthermore, PD patients over-weight degraded visual cues (Yakubovich et al. 2020), indicating resistance in certain sensory pathways. A key clinical implication is that improvements in motor control may not always translate to restored perceptual-motor integration (Ding et al. 2017; Waldmann et al. 2020; Yakubovich et al. 2020), leaving patients vulnerable to disorientation or altered body perception. This duality necessitates a nuanced and individualized approach to medication management. Future work should adopt unified experimental paradigms that compare ON vs. OFF dopaminergic states across various sensory channels to delineate the boundaries of dopamine's influence on multisensory processing in PD.

### Limitations, implications, and future directions

#### Sources of methodological heterogeneity

While the reviewed literature consistently demonstrates MSI deficits in PD, substantial methodological heterogeneity prevented a formal meta-analysis. Several key factors contributed to the variability in the reported magnitude and nature of these deficits:

*Medication State Variability.* Testing states varied significantly across the 40 studies, with 24 assessing patients exclusively in the ON-medication state, 7 in the OFF state, and 8 comparing both. Because dopaminergic therapy influences sensory processing unevenly, this creates conflicting data. For instance, while medication often improves muscle strength and basic gait mechanics, it does not normalize proprioceptive reweighting or temporal binding impairments, and may even increase susceptibility to the RHI. Consequently, studies testing exclusively in the ON state may underreport baseline MSI dysfunction, while those combining ON and OFF state data introduce confounding variables regarding dopaminergic responsiveness.

*Disease Severity and Staging.* Although most studies focused on mild to moderate PD (Hoehn and Yahr stages I–III), the methods for establishing this baseline differed. Staging was assessed in the ON state in some cohorts and the OFF state in others, drastically reducing direct comparability. Furthermore, the inclusion of more advanced cases (stages III–IV) in a minority of studies introduces participants who likely suffer from broader neurodegeneration and cognitive decline, complicating the isolation of pure MSI deficits from general disease progression.

*Cognitive Function and Exclusion Criteria.* MSI tasks, particularly audiovisual semantic integration and temporal binding, require baseline cognitive resources. Studies utilized varying cognitive exclusion criteria (e.g., MoCA cut-offs ranging from 21 to 25, or differing MMSE parameters), while some excluded cognitive dysfunction entirely to ensure homogeneity. This discrepancy means that some studies likely captured MSI deficits intertwined with mild cognitive impairment (MCI), whereas others isolated purely sensory-level deficits, contributing to varied performance outcomes across similar paradigms.

*Task Paradigm and Sample Size. Constraints* The paradigms used to measure MSI varied from motor-centric balance assessments (e.g., SOT) to purely perceptual illusions (e.g., RHI, Sound-induced Flash Illusion). Comparing a perceptual threshold to a motor reflex introduces significant task-dependent heterogeneity. Additionally, smaller sample sizes in several highly specialized neuroimaging or perceptual studies may have resulted in underpowered analyses, potentially skewing effect sizes when compared to larger, clinically focused motor evaluations.

#### Clinical implications

A comprehensive understanding of PD requires acknowledging the interconnected nature of multisensory, motor, and perceptual systems. MSI deficits disrupt a patient's ability to maintain equilibrium and their perception of their own body, which in turn complicates motor coordination and increases fall risk. Therefore, addressing these deficits requires a holistic approach that targets both the motor symptoms and the underlying sensory and perceptual disruptions.

*Refining clinical practice and assessments.* To incorporate MSI evaluation into routine clinical practice, traditional motor-centric evaluations (e.g., MDS-UPDRS) should be complemented with targeted sensory-conflict assessments. In standard clinical settings without access to specialized equipment, this can be achieved by using low-cost clinical derivatives of the SOT, such as the Modified Clinical Test of Sensory Interaction on Balance (mCTSIB) (Shumway-Cook & Horak 1986; Harro et al. 2016). By assessing patients as they stand on a compliant surface (foam) with their eyes closed, clinicians can easily remove reliable visual and somatosensory cues. This provides a highly sensitive, functional measure of cognitive-sensory integration and vestibular reliance. Additionally, simple visual-proprioceptive assessments derived from experimental paradigms, such as arm-matching tasks without visual feedback (Adamovich et al. 2001; Rabin et al. 2010), can help clinicians explicitly unmask visual dependency and identify subclinical MSI deficits. In specialized clinics, assessments can be scaled up to include advanced visual perturbation tasks using portable movement monitors (wearable inertial sensors) or dynamic VR-based platforms (Canning et al. 2020; Mirelman et al. 2013). These tools enable clinicians to objectively quantify how a patient's balance and spatial cognition deteriorate under sensory conflict, leading to highly targeted fall-prevention strategies.

*Enhancing Rehabilitation Strategies.* Therapeutically, MSI findings advocate for the development of tailored rehabilitation interventions that directly address sensory integration deficits. This includes adapted physical therapy protocols that explicitly challenge and train MSI capacities, such as sensory reweighting exercises where patients learn to safely prioritize proprioceptive feedback over degraded visual cues (Nocera et al. 2009). Furthermore, VR-based training programs offer highly controlled environments to simulate sensory conflicts, proving particularly effective in helping patients improve motor control and balance under complex, real-world sensory conditions (Canning et al. 2020; De Natale et al. 2025).

*Enhancing Rehabilitation Strategies.* Therapeutically, MSI findings support the development of tailored rehabilitation interventions that directly address sensory integration deficits. This includes VR-based training programs that integrate multiple sensory cues to improve motor control and balance (Canning et al. 2020; De Natale et al. 2025) and adapted physical therapy protocols that explicitly challenge and train MSI capacities (Nocera et al. 2009). Strategies like sensory reweighting and VR-based training are particularly effective in helping patients improve their response to various sensory inputs.

*Personalized Pharmacological Strategies.* Preliminary studies indicate that dopaminergic medication can differentially affect MSI, particularly visual–vestibular processing and postural stability, sometimes producing detrimental effects (Cham et al. 2007; Ding et al. 2017; Mongeon et al. 2009; Müller et al. 2019). This highlights the need for personalized pharmacological strategies, including dosage adjustments or alternative therapies, to balance motor symptom relief with sensory–perceptual outcomes.

*MSI Dysfunctions as Functional Biomarkers.* MSI dysfunctions may offer functional biomarkers of disease progression. For instance, prolonged audiovisual TBW (Rostami et al. 2024) may serve as early-stage indicators. Evidence that altered multisensory temporal processing can precede prominent motor symptoms and is associated with cortical thinning (Zhou et al. 2025) further supports their potential as biomarkers for early detection and monitoring of disease progression. In genetic cases of PD (e.g., LRRK2 or GBA mutations), stratifying patients by MSI profile could help characterize subtypes and tailor treatment, improving the understanding of PD heterogeneity and pathophysiology (Dulski et al. 2022).

#### Strengths and limitations

Research on MSI in PD highlights both the strengths and limitations of current studies. Strengths include the detailed examination of audiovisual, visual-tactile, proprioceptive, and visual-vestibular integration using various methodologies, such as reaction time tasks, RHI, and virtual reality-based assessments. These studies consistently demonstrate that PD patients exhibit impairments in MSI across multiple sensory modalities, often with specific deficits in temporal processing, spatial accuracy, and sensory reweighting. Notably, the involvement of the subthalamic nucleus and the role of cholinergic deficits are recurrent themes, emphasizing their significance in MSI.

Beyond the methodological heterogeneity discussed above, a significant limitation is the frequent exclusion of participants with cognitive impairment, which reduces the ecological validity of the findings and severely limits insight into the full clinical spectrum of PD. Even when cognitive function is assessed, some studies rely on tools like the MMSE, which is insensitive to typical PD-related deficits (e.g., executive and visuospatial dysfunction), potentially overlooking mild cognitive impairment. Additionally, most studies focus on isolated sensory modalities, rather than a truly multimodal approach that better reflects real-world processing. While many studies explore the underlying neural mechanisms, the clinical translation of these findings into effective interventions remains underexplored. These methodological inconsistencies pose threats to validity, particularly in the form of selection bias, measurement bias, and confounding.

#### Future directions

Future research in PD should focus on several key areas to enhance our understanding and treatment of MSI deficits. Firstly, investigating the efficacy of MSI therapies is crucial; studies could explore how targeted interventions, such as VR-based therapies or sensory reweighting exercises, can improve both motor and sensory outcomes (Freeman et al. 2018; Hawkins et al. 2021). Secondly, further research into the role of specific neurotransmitter systems, such as the cholinergic system and its impact on MSI (Bohnen et al. 2022a; Müller et al. 2013), could provide insights into how neurotransmitter imbalances contribute to sensory deficits. Finally, developing more sophisticated diagnostic tools that capture the multifaceted nature of MSI deficits in PD is essential. Innovations such as advanced neuroimaging techniques or real-time sensory integration assessments could lead to earlier and more accurate diagnosis, facilitating timely and personalized interventions (Ding et al. 2017, 2018; Waldmann et al. 2020). The emerging role of AI and large language models (LLMs) in analyzing complex sensory data and patient profiles offers unprecedented opportunities to refine diagnostics, personalize interventions, and predict disease progression, moving towards truly individualized and proactive patient care in PD.

## Supplementary Information

Below is the link to the electronic supplementary material.

Supplementary material 1

## Data Availability

No new datasets were generated or analysed during this study. All data referenced in this review are from previously published studies and are available in the public domain through their respective sources.
