# Supplementary material for: Beyond motor: a systematic review of multisensory integration deficits in Parkinson’s disease
Source: J Neural Transm (Vienna). 2026 May 29;133(7):1069–85. doi: 10.1007/s00702-026-03184-2 (PMC13428709; doi:10.1007/s00702-026-03184-2)
Supplement: Supplementary file 1 — Supplementary material 1 [file 702_2026_3184_MOESM1_ESM.pdf]

Appendix 1

Table 1: Studies (n=40) investigating MSI in the people with PD rated with the Newcastle-Ottawa Quality Assessment Scale (NOS)(Wells et al., 2000).

	**Study**	**Quality assessment criteria**								
		Selection (4 stars)				***Comparability (2 stars)***		***Exposure (2 stars)***		***Quality score (Maximum = 8 stars)***
		Is the population definition adequate?	Representativeness of the population	Selection of controls	Definition of controls	Study controls for age	Study controls for additional factor	Same method of ascertainment for cases and controls	Outcome Measures	
		Individuals with PD	Representative of individuals with PD (covering all stages)	Same community	No current severe or chronic disease	Individual with PD to age-matched older adults	Are the populations in the studies comparable in terms of cognitive abilities?	Methods are identical	Same outcome for both groups	
1	Rostami et al., 2024	*	-	*	*	*	*	*	*	7
2	Tran et al., 2023	*	-	*	*	*	*	*	*	7
3	Bohnen et al., 2022a	*	*	-	*	*	*	*	*	7
4	Bohnen et al., 2022b	*	*	-	*	*	*	*	*	7
5	Hawkins et al., 2021	*	-	*	*	*	*	*	*	7
6	Waldmann et al., 2020	*	-	*	*	*	*	*	*	7
7	Ding et al., 2018	*	-	*	*	-	*	*	*	6
8	Freeman et al., 2018	*	-	-	*	-	-	-	-	2
9	Ren et al., 2018	*	-	*	*	*	*	*	*	7
10	Ding et al., 2017	*	-	*	*	*	*	*	*	7
11	Fearon et al., 2015	*	*	-	*	*	*	*	*	7
12	Muller et al., 2013	*	-	-	*	*	*	*	*	6

13	Barnett-Cowan et al., 2010	*	-	*	*	*	*	*	*	7
14	Rabin et al., 2010	*	-	*	*	*	*	*	*	7
15	Sabate et al., 2008	*	*	*	*	*	*	*	*	8
16	Hwang et al., 2016	*	-	-	*	-	-	-	-	2
17	Harrington et al., 2014	*	-	*	*	*	*	*	*	7
18	Sol Yakubovich et al., 2020	*	-	*	*	*	*	*	*	7
19	Huh et al., 2016	*	-	*	*	*	*	*	*	7
20	Nallegowda et al., 2004	*	-	*	*	*	-	*	*	6
21	Harro et al., 2016	*	*	-	*	*	*	*	*	7
22	Gera et al., 2016	*	*	-	-	*	-	-	-	3
23	DiFrancisco-Donoghue et al., 2015	*	-	-	*	*	-	*	*	5
24	Chong et al., 1999	*	*	*	*	*	*	*	*	8
25	Colnat-Coulbois et al., 2011	*	-	-	*	*	-	*	*	5
26	Sadeghi et al., 2024	*	-	*	*	*	*	*	*	7
27	Feller et al., 2019	*	-	*	*	*	-	*	*	6
28	Bek et al., 2022	*	*	-	*	*	*	*	*	7
29	Scarpina et al., 2019	*	-	*	*	*	*	*	*	7
30	Jose Luvizutto et al., 2020	*	-	*	*	*	*	*	*	7
31	Harro et al., 2018	*	-	-	*	-	-	-	-	2
32	Adamovich et al., 2001	*	-	*	*	*	*	*	*	7
33	Scarpina et al. (2019b)	*	-	*	*	-	*	*	*	6
34	Konczak et al. (2012)	*	-	*	*	*	*	*	*	7
35	Brown et al. (2006)	*	-	*	*	*	-	*	*	6
36	Copland et al. (2000)	*	-	*	*	*	-	*	*	6
37	Zhou et al. (2025)	*	-	*	*	*	*	*	*	7
38	Shoji et al. (2024)	*	-	*	*	*	-	*	*	6

39	Vervoort et al. (2013)	*	-	*	*	*	*	*	*	7
40	Honma et al. (2018)	*	-	*	*	*	*	*	*	7

(*) = Acceptable; (-) = Not acceptable
